# “Sometimes we can’t fix things”: a qualitative study of health care professionals’ perceptions of end of life care for patients with heart failure

**DOI:** 10.1186/s12904-016-0074-y

**Published:** 2016-01-14

**Authors:** Margaret Glogowska, Rosemary Simmonds, Sarah McLachlan, Helen Cramer, Tom Sanders, Rachel Johnson, Umesh T. Kadam, Daniel S. Lasserson, Sarah Purdy

**Affiliations:** Nuffield Department of Primary Care Health Sciences, University of Oxford, Radcliffe Observatory Quarter, Woodstock Road, Oxford, OX2 6GG UK; Centre for Academic Primary Care, NIHR School for Primary Care Research, School of Social and Community Medicine, University of Bristol, Canynge Hall, 39 Whatley Road, Bristol, BS8 2PS UK; Arthritis Research UK Primary Care Centre, Research Institute for Primary Care & Health Sciences, Keele University, Staffordshire, ST5 5BG UK; Section of Public Health, ScHARR, University of Sheffield, 30 Regent Street, Sheffield, S1 4DA UK; Health Services Research Unit, Innovation Centre 2, Keele University, Staffordshire, ST5 5NH UK

**Keywords:** End of Life care, Palliative care, Advance care planning, Heart failure

## Abstract

**Background:**

Although heart failure has a worse prognosis than some cancers, patients often have restricted access to well-developed end of life (EoL) models of care. Studies show that patients with advanced heart failure may have a poor understanding of their condition and its outcome and, therefore, miss opportunities to discuss their wishes for EoL care and preferred place of death. We aimed to explore the perceptions and experiences of health care professionals (HCPs) working with patients with heart failure around EoL care.

**Methods:**

A qualitative in-depth interview study nested in a wider ethnographic study of unplanned admissions in patients with heart failure (HoldFAST). We interviewed 24 HCPs across primary, secondary and community care in three locations in England, UK – the Midlands, South Central and South West.

**Results:**

The study revealed three issues impacting on EoL care for heart failure patients. Firstly, HCPs discussed approaches to communicating with patients about death and highlighted the challenges involved. HCPs would like to have conversations with patients and families about death and dying but are aware that patient preferences are not easy to predict. Secondly, professionals acknowledged difficulties recognising when patients have reached the end of their life. Lack of communication between patients and professionals can result in situations where inappropriate treatment takes place at the end of patients’ lives. Thirdly, HCPs discussed the struggle to find alternatives to hospital admission for patients at the end of their life. Patients may be hospitalised because of a lack of planning which would enable them to die at home, if they so wished.

**Conclusions:**

The HCPs regarded opportunities for patients with heart failure to have ongoing discussions about their EoL care with clinicians they know as essential. These key professionals can help co-ordinate care and support in the terminal phase of the condition. Links between heart failure teams and specialist palliative care services appear to benefit patients, and further sharing of expertise between teams is recommended. Further research is needed to develop prognostic models to indicate when a transition to palliation is required and to evaluate specialist palliative care services where heart failure patients are included.

## Background

Heart failure is a life-limiting condition [1], associated with high morbidity [2]. It places a significant burden on NHS resources, accounting for 2 % of inpatient bed-days and 5 % of emergency medical admissions to hospital [3]. The trajectory of the illness varies but a general pattern is discernible [4, 5]. Patients may experience a phase of stable, although functionally relatively poor, health. After this stable phase, a period of decline may ensue, interspersed with exacerbations, requiring increased use of secondary care, and remissions of their heart failure. Death may occur as a natural progression or suddenly and unexpectedly, especially among older people [1].

While heart failure has a worse prognosis than some cancers [6], there is evidence that patients with advanced heart failure have a poor understanding of their condition and its outcome [7, 8] and that few patients are given the opportunity to discuss their end of life (EoL) care, including their preferred place of death, with clinicians [9, 10]. This contrasts with the experience of cancer patients who often have better access to information about their illness and to well-developed models of care [11, 12], but is similar to that of patients with other non-malignant, life-limiting conditions, especially chronic obstructive pulmonary disease (COPD) and motor neurone disease (MND) [13–15].

Palliative care services originally developed around cancer patients, alongside more limited development for patients with non-malignant neurological disease. Typically, patients with non-malignant conditions have had less access to these services. There has been growing recognition of what palliative care services can offer patients with other terminal conditions and calls for improved access to specialist palliative care services for heart failure patients [10, 16, 17]. However, more recent research suggests that the change of focus from ‘active’ treatment to palliative care is not being managed among heart failure patients, that patients’ needs for co-ordinated, generalist EoL care are still not being met and that their access to specialist palliative care services remains limited [18, 19].

In this paper we explore the experiences and perceptions of health care professionals (HCPs) across primary care, secondary care and community settings of the EoL care available for their heart failure patients and their role within it. We were interested in exploring the extent to which HCPs believed patients’ needs were being met at EoL and whether barriers to accessing appropriate care and support still exist for patients with heart failure as they approach death.

## Methods

### Sampling and data collection

The HCP interviews took place in three geographical locations in the UK where people with severe or difficult to manage heart failure were participating in a wider study (HoldFAST), a multi-centre ethnographic study of unplanned hospital admission for heart failure [20]. The research was approved by the NHS Health Research Authority Research Ethics Committee South West (reference 12/SW/0104). Purposive sampling was employed to select a range of HCPs caring for people with heart failure across primary, secondary and community care settings (see Table 1).

**Table 1:** HCPs who took part in the interviews

Staff based in primary care	Staff based in secondary care	Staff based in community care
Location 1 South West		
GP [P1] GP [P2] GP [P3] GP (interviewed twice) [P4]	Cardiologist [P8] Care of the elderly physician [P9] Hospital liaison psychiatrist [P10]	Specialist heart failure nurse [P20] Community matron [P21]
Location 2 South Central		
GP [P5] GP [P6]	Cardiologist [P11] Specialist heart failure nurses (interviewed together) [P12 and 13] HF specialist nurse [P14] Cardiac Rehabilitation manager[P15]	Specialist heart failure nurses (interviewed together) [P22 and 23]
Location 3 Midlands		
GP [P7]	Cardiologist [P16] Specialist heart failure nurses) (interviewed together) [P17 and 18] Cardiac Rehabilitation practitioner [P19]	Specialist heart failure nurse [P24]

Individual, in-depth interviews mainly took place in the HCPs’ workplaces. The HCPs were given information about the study and had chance to ask questions. Written informed consent was obtained from all participants. We used a topic guide to ensure coverage of areas of relevance to the research questions but also allowed the participants to raise their own issues. The majority of the interviews (20) were recorded digitally and transcribed verbatim; the remainder (2) were written up as field notes.

### Data analysis

The analysis process was informed by a ‘grounded theory approach’ [21] and the constant comparative method [22], which included familiarisation with the transcripts and notes, followed by detailed open coding using NVivo 10 qualitative data analysis software. We generated an initial coding framework from the first set of interviews in one of the locations. We then examined the two sets of interviews from the other locations, adding to and re-organising the coding framework until we reached a final set of codes. The codes were then refined and built into wider categories, taking into account confirming and disconfirming data and similarities and differences across the three locations. The categories were then checked against all the transcripts to ensure that all material had been included. The research team discussed the coding framework as analysis progressed, the ideas for the categories which emerged from the data and the final themes to ensure their credibility and confirmability [23, 24]. At this point, we invited our patient/carer advisory group to join with the research team in reviewing the full set of themes and contributing to final interpretations of the data.

## Results

Our study identified three major themes in the HCPs’ accounts around EoL issues among patients with heart failure. The first of these was how death and dying are brought to the attention of patients and their families. Secondly, professionals discussed the issue of recognising when patients might be at their EoL. Thirdly, the professionals’ attention was focused on the frequent hospital admissions of patients at the end of their lives, and their experience of working with services which might provide alternatives to admission.

### Having ‘the conversation’: raising the issue of death and dying

The HCPs expressed concerns about the extent to which patients and their families understood heart failure and when they might be nearing death. They acknowledged that professionals’ reluctance to communicate information to patients and families could restrict opportunities to have ‘the conversation’. They recognised that some patients would never accept that they were approaching death:

*“…some patients are never ready, never ever ready to have that conversation”* [P21, community matron]

Others who had undergone previous successful interventions might be reluctant to accept that nothing further could be done:

*“You’ve got to remember if they’ve come from an MI* (myocardial infarction) *point of view, every time they’ve had a problem it’s been fixed, they’ve either had a stent put in…or bypass, so things have been sorted.”* [P20, community specialist heart failure nurse care]

Situations were recounted by the HCPs where family members sought to hide the truth and protect each other in the face of bad news:

*“…the carers will say please don’t tell the patient and you have this rather awkward scenario where the patient knows that things are not right, and the carer knows that things are not right, but neither wants the other to know…they don’t want the children to worry or the children don’t want the parents to worry…”* [P11, cardiologist]

There was consensus that being honest with patients was the right, ethical course to take and less likely to provoke conflict within families:

*“Well towards the end of your life you do not want to be playing games with your nearest and dearest…you want to be able to talk quite freely…and in the end there is much less upset I think from people being honest.”* [P9, care of the elderly physician]

The participants expressed views on which professionals were best placed to have ‘the conversation’ with patients. One GP valued the input of specialist heart failure nurses in this regard. A community matron also perceived that the relationships she developed with patients meant that she could more easily raise the topic of death:

*“…the GPs are amazing with their medicine management…but it’s all the other bits, the fear, the frightened, when somebody touches your hand and says ‘am I going to die?’, you know that’s not a conversation you can have in a consulting room in eight minutes, you know that’s conversations that happened because they trust you enough to say I’m frightened, you know.”* [P21, community matron]

One of the community-based nurses explained that getting to know patients enabled her to introduce the topic so that patients had time to think:

*“…it isn’t that often that we’re involved only at the end of life, usually we’ve known the patient for a while and we’ve been able to start having end of life conversations quite a while before it is actually the end of life…and if you try and plant the seed a bit, and just try and make patients and the family think a little bit more about end of life and things they might wish and it’s interesting how you think a patient is in possibly in a bit of denial about it, but actually when you get them one to one, they have often been thinking about all these things and they welcome the fact that you’ve opened that up to them and they can talk about it a bit more.”* [P23, community specialist heart failure nurse]

Thus, there was an understanding that discussions could best happen in the context of an established relationship:

*“And then if a patient continues to deteriorate it’s helpful for the family and for the patients to have some expectation that things might be slowly worse, and also then that difficult discussion about what, where do they want to be looked after, where do they want to be cared for, and what sort of care do they want at the end when that happens? And that will be during the course of that relationship with that patient. I wouldn’t dive in and talk about all that initially.”* [P4, GP]

The HCPs’ accounts indicate their wish to be honest with patients and their belief that patients had a right to expect this. They would not be deterred by patients seeming unresponsive to their messages but would continue to raise the topic:

*“And the ones, the patients that do go into the palliative phase more often than not know that they’re going into the palliative phase, they themselves will recognise the fact that they are now actually dying and are quite happy to talk to you about where they want to go to die and the things that we can put in place to make them comfortable…I think we have a responsibility to keep trying to discuss this issue because otherwise that person we know is going to end up in a crisis situation and we’re trying to avoid that as much as we possibly can.”* [P18, hospital specialist heart failure nurse]

One GP claimed that patients may not be concerned so much with the fact that they will die but the manner in which it will happen. This participant explained how a patient’s visit to a hospice had provided reassurance:

*“…just getting used to the fact that, you know, what will happen will happen…but actually he’s unlikely to suffer etc. and I think maybe that was reassuring for him.”* [P4, GP]

### “Ringing warning bells”: recognising EoL

A frequently mentioned factor in the failure to start ‘the conversation’ was the uncertainty about the trajectory of the illness and difficulty in predicting when patients might be entering the terminal phase of the condition. Commonly, patients undergo episodes of exacerbation before they die, engendering expectations in themselves and their families that they will always recover*:*

*“…they’ve said to him five times before now he’s not going to get out of hospital and each time he bounces back and…you just know that one time they’re not going to bounce back and that’s going to come as a huge, huge blow.”* [P13, hospital specialist heart failure nurse]

Another participant summed up the dilemmas faced by the family of a patient, who on previous occasions had been close to death but had survived each time:

*“Her family have really struggled with end of life issues in particular – when is she at her end of life?”* [P6, GP]

Thus, the timing of any conversation about planning for EoL care calls for careful consideration. One of the HCPs considered the competing issues – scaring patients and taking away their hope *versus* providing some warning that death may occur:

*“…you don’t want to frighten people unnecessarily or cause unnecessary distress, but on the other hand people deserve a sort of warning that this might be the case and you don’t want to take away people’s hope, you know it’s a very difficult area with heart failure, discussions about end of life.”* [P12, hospital specialist heart failure nurse]

Similarly, another HCP weighed up the risk of shocking patients by discussing EoL care against ignoring the issue but then struggling to make suitable provision in a crisis situation:

*“But anyway there is forever that tension between getting in early enough for it to be nice and smooth and orderly, versus…but at the risk of shocking people by increasing their awareness of their imminent death versus leaving it and then sort of scrambling through the process when you are in a crisis.”* [P6, GP]

Clinicians across the settings highlighted features which they would consider indicative of patients approaching death. These included hospitalisations occurring closer together, exhausting treatment options, intolerance of current treatments and worsening renal function:

*“…in those patients that are having those episodes closer together that’s when we may be having those discussions with regards to what they would like, a preferred place of care should this disease go into the palliative phase because a good indicator that they are going into the palliative phase is that these episodes get more frequent and closer together so that tends to be the pattern…with worsening renal function and things like that.”* [P17 and 18, hospital specialist heart failure nurses]

*“…you’ve had a gentleman that’s on his third admission in six months with heart failure, well shouldn’t that be ringing warning bells…that this gentleman is not getting any better with treatment that we’ve started him and actually we should be having those conversations with him about what does he expect in the future…”* [P14, hospital specialist heart failure nurse]

The HCPs’ accounts also provided examples where they perceived the necessary discussions had not taken place and where patients in advanced heart failure had been aggressively treated for infections, instead of receiving palliative support:

*“I…thought this patient is dying…so I spoke to the consultant and said can you make this patient not for resus*[citation]*…his opinion was you’ve got to get better, you’ve got to fight this illness…I said to the patient I think your symptoms are quite a lot, you’re quite nauseous because I can tell by your, the way that you’re acting. He said I feel really really sick. So I called palliative care in and he did die…he had a false message, so I was trying to take it down the palliative route because I thought that was appropriate…but the clinician wanted to treat an infection…”* [P20, community specialist heart failure nurse]

There was concern that lack of communication had consequences for choosing appropriate treatment and care:

*“We had a situation with a patient who was end stage and we said to the doctor straight away this chap’s end stage…shouldn’t we be thinking palliative should get involved, and they treated him for cellulitis, and said no, no we’re going to treat him for that…you could see this chap was dying, going to die in hospital if somebody didn’t do something…nobody’s making the decisions here, the doctors are leaving it up to the patient, the patient is unwell, confused and can’t make that decision for himself…It was really frustrating and I think that happens a lot.”* [P14, hospital specialist heart failure nurse]

Participants considered that professionals should recognise the situations where it would not be possible to make patients well again:

*“…nobody has those conversations with patients in hospital, like have you thought about the future, have you put a will in place…medics like to fix things and sometimes we can’t fix things.”* [P14, hospital specialist heart failure nurse]

### Avoiding the “default” setting: EoL care and hospital admissions

The HCPs accepted that heart failure patients at the end of their lives were repeatedly hospitalised, even when no further interventions would change the course of the condition, because of the lack of planning and provision that could keep them in the community. In location 2, this was attributed to patients being admitted by out of hours doctors who did not know them:

*“Unfortunately I don’t think we have very well developed mechanisms for keeping them in the community in the latter stages of their illness, and this is all due to a whole series of issues…It’s due to the fact that often when they become more unwell they are seen by a primary care physician who doesn’t know them…if it’s on-call services and therefore will default to admission to hospital…”* [P11, cardiologist]

Two GPs recounted situations where patients, whom they would have preferred to manage at home, had been admitted:

*“…people who’ve already reached sort of the end of life care stage, who if I’d have been called out knowing that that’s the situation, I might have managed the situation very differently. Somebody who doesn’t know them then might admit them because they’re in extremis, whereas in fact they may well have previously said they don’t want to be admitted in that situation or the family might have accepted that they’re going to die anyway so keep them at home and keep them comfortable.”* [P5, GP]

*“…given her advanced stage and multiple conditions and the fact that I had already talked with her family and her about the fact that life wasn’t going to go on indefinitely…I still think I would have preferred to have managed her at home.”* [P6, GP]

Other factors included families struggling to manage patients at home or patients living alone without sufficient support:

*“…it may be pressure from the relatives who feel that they can’t cope with the patient at home, or it may be simply that the patient is alone at home and it is not possible to provide the level of support necessary at home to keep them at home when they’re immobile and ill so I think there’s a big issue with that side of the management of this condition…”* [P11, cardiologist]

However, there was agreement that hospital was not the best place to die and that, with appropriate provision, most people would choose to be at home:

*“…we want more people to not go into hospital towards the end of their lives unless it’s something that can only be managed in hospital and we want people to die where they want to which is usually at home and we want it to be a much nicer and better death than is often the case.”* [P6, GP]

The HCPs recognised the importance of advance care planning if patients were to die at home and described actions they were taking, or considering, that might enable this to happen:

*“I try and leave a printout, an up-to-date sort of summary printout with such patients so that if they get a visit from the Out of Hours…they will have much more information available to them…it’s usually when things are coming to some sort of a head that we do let them know, particularly when we think somebody is going to die, they said specifically they wanted to die at home we have got things set up and we are really trying to stop a hospital admission occurring…there seems to be such a…sort of default position to admit people if they have acutely deteriorated.”* [P6, GP]

*“And at the moment we have a palliative care register, I think pretty much everybody on it at the moment has got a malignancy and they meet every three months, but actually I think we need to be considering people with heart failure…people who are going to get worse over time and they’re going to need more care, and if it helps to provide, if it helps to have more regular meetings with a wider multidisciplinary team.”* [P4, GP]

There was recognition of the increasing role that specialist heart failure nurses and community matrons could play in supporting patients at home:

*“…it is something that we are very involved in* [end of life care]*, and even since I first started it must have been exactly the same with patients with heart failure they were getting unwell and becoming palliative and end stage but it’s become a lot more kind of part of our job in the last two or three years…”* [P23, community specialist heart failure nurse]

There was also acceptance that they could not provide round-the-clock services and that symptoms like breathlessness required careful management in the community:

*“I do find with heart failure and COPD in the end stages, because of the shortness of breath in both, the heart failure and the COPD, it’s dreadful, it’s very difficult to manage, very difficult medically to manage, they need lots and lots of support around them all the time and that’s something the community can’t do, we can’t provide 24 hour care…”* [P21, community matron]

Both primary and secondary care physicians acknowledged that they were unsure about what services were available or that there were few services upon which they could call:

*“…it’s sometimes confusing as to exactly who would be best placed to help the patient.”* [P6, GP]

*“…there are various home methods of looking after people with even quite bad heart failure…you can put diuretics in a syringe driver for instance…so the person could stay at home if oedema’s the problem…and there are numerous things like that. It depends really what the patient wants to some degree and also what’s available locally. I’m afraid our trust does not have a great deal to offer.”* [P9, care of the elderly physician]

One GP advocated the extension of palliative care to heart failure and voiced the need for good communication between secondary care and primary care so that primary care could be alerted when the emphasis for patients should shift from active treatment to palliation:

*“…providing some hand holding for primary care clinicians like myself to say ‘no, all the treatment has been given that can be given’…I think there needs to be very good communication between secondary care and primary care to identify when palliation is required.”* [P3, GP]

Across the locations, links between heart failure services, in both hospital and community settings, and specialist palliative care services had been established. In particular, the specialist heart failure nurses were able to liaise closely with, and refer patients to palliative care services. However, in location 2, the uncertain course of heart failure made it difficult to judge when to put in place palliative care support, which could be offered on a time-limited basis only:

*“…the difficulty with end of life care and heart failure is the unpredictability of the time scale and so getting palliative care services involved and the sort of end of life package which you can get, which is very intensive and very good, is only for a short amount of time, and if you think they might live longer than that…they’re probably saying well it’s too soon for us to get involved…”* [P12, hospital specialist heart failure nurse]

In all three locations, hospices admitted heart failure patients, although their capacity was limited. They also provided support and outreach to heart failure patients in advance, in the form of provision such as a breathlessness clinic and day visiting. GP participants in two of the locations described individual patients who were receiving care from a hospice. One patient had established links with a local hospice and was attending their day centre weekly. Another had been admitted to the hospice when very unwell and while he had made some recovery, focus for him had shifted from active intervention to palliative care:

*“…so the hospice were great because they took him out of the home scene and looked after him, stopped much of his medication and put him on an anti-depressant. So he actually recovered quite well really…I think just being out of that, the home environment and having some time, sort of respite for himself really, helped no end.”* [P4, GP]

For both these patients and their families the care provided at the hospice had provided important respite.

## Discussion

The study identifies three main issues which still significantly affect the EoL care available for patients with heart failure. Our multi-centre, multi-disciplinary data highlighted the continuing lack of attention paid to EoL care for patients with heart failure across locations in the UK.

### Raising the issues of death and dying

The HCPs’ accounts reveal the perceived gap between how they would choose to approach ‘the conversation’ with patients and their families and the reality of introducing sensitive issues [7, 25]. Societal attitudes towards death [26] are not perceived as conducive to open dialogue, making the professional’s task considerably harder. Timing was perceived to be a major consideration in raising issues of death and dying. The HCPs acknowledged the tension between introducing the topic early on at a point when death is unlikely to occur and active treatments are ongoing *versus* organising a timely referral to palliative care services when death appears more likely. Greater integration of palliative care services into heart failure services may facilitate earlier discussions as well as improve symptom control at an earlier time point along a declining clinical trajectory.

GPs and community-based nurses, who already had a relationship with the patients concerned, perceived themselves as able to introduce sensitive subjects, although nurses saw themselves as having more time to do this. In this way, ‘the conversation’ was likely to become ‘the conversations’ – an ongoing process rather than a single event, where patients could discuss dying while they had capacity and could be in control [27]. As research suggests, patients may be more open to discussions about planning EoL care than doctors imagine [28, 29]. The participants believed patients would benefit from communication, would welcome opportunities to discuss EoL provision and seek reassurance that they would not suffer [30]. Their accounts indicated that patient preferences are not easy to predict and that sensitivity to the wishes of the individual regarding advance care planning is paramount [31].

### Recognising EoL

While uncertainties around predicting EoL militated against ‘the conversation’ taking place, the HCPs highlighted circumstances that should trigger discussions around EoL care. Consistently mentioned as a sign of decline was more frequent hospitalisation, particularly for episodes of exacerbation, consistent with other studies [5, 6, 16, 19] – the so-called revolving door patients [32]. Such an event is comparable with findings elsewhere and may be an indicator of a transition point [19], at which emphasis on palliative rather than restorative treatment is appropriate. The Gold Standards Framework Prognostic Indicator Guidance [33] aims to identify patients in their last year of life (using a set of simple criteria, including the ‘surprise question’), thereby prompting clinicians into initiating discussions with patients around EoL issues. Very limited evidence of its accuracy is available [34, 35]. Similarly, clinical indicators and biochemical markers, such as the need for intravenous therapies and measures of cardiac performance, which indicate the onset of the terminal phase of heart failure, are documented in the literature [5, 19], but there is a dearth of prognostic models which would help aid clinicians’ decision-making [10, 18, 34].

Because of the uncertainties around prognostication and the barriers to communication, several of our participants identified situations in which clinicians struggled to recognise and inform patients that they had reached the EoL stage and plan care appropriately – a state described as prognostic paralysis [4, 30, 36, 37]. Such situations are borne out in the literature which suggests that doctors may not always recognise the closeness of death [19], may have a ‘treatment imperative’ making it difficult for them to accept that heart failure is not a problem to be *“fixed”,* [27] may be over-optimistic as to what can be achieved through intervention [38] or may even perceive the death of a patient as a failure of the health care received [26]. Thus patients may actually end up receiving major interventions shortly before their death [39].

### EoL care and hospital admissions

Across all our study participants there was acceptance that hospital admission for patients in the terminal phase of heart failure – the *“default”* setting – was widespread. In one location, participants perceived this to be linked to the organisation of out of hours services, geared more to managing acute emergency situations, thus making it more likely that patients would die in hospital than at home [14, 40]. While patients may now have more access to holistic support at home, in the form of specialist heart failure nurses and community matrons, which might reduce admissions, few patients will have discussed place of death or wishes for EoL care in advance [9, 41].

The community-based nurses explained how caring for patients at the end of their life has become increasingly important in their role and studies have concluded that nurses possess the skills necessary to care for patients at EoL, namely communication, all-round care focused on the patient and attention to controlling symptoms [32, 38]. Specialist nurses are considered to be proactive participants in providing quality EoL care [42] and there is acknowledgment among specialist palliative care services that they are already fulfilling generalist palliative care roles [19]. In each location, clinicians had established links with specialist palliative care services and small numbers of individual patients were accessing their care and support [5]. Where arrangements were in place, these were viewed positively by the participants, who were eager to find ways of working with specialist palliative care services [43, 44].

However, significant barriers still exist which limit the provision of specialist palliative care for heart failure patients [18, 45]. The literature demonstrates that the palliative care needs of heart failure patients are less well understood than those with malignant disease [46] and there is limited evidence to date of the worth of palliative care for life-limiting conditions such as heart failure [47, 48]. Among our participants the unpredictable trajectory of the condition contributed significantly to clinicians’ uncertainty about when to initiate a palliative approach to care, commensurate with other non-malignant conditions [49–51].

### Strengths and limitations of the study

We interviewed HCPs – including seven GPs, 10 specialist staff members based in secondary care and five community staff members – across varied UK settings and were able to gain an overview of what part primary, secondary and community care services play in provision of EoL care and how they interface with specialist palliative care services. The use of in-depth interviewing as a methodology gave our participants the opportunity to talk freely about their experiences of EoL care for heart failure patients, responding to our questions about how services were delivered and offering their own insights into how care was provided and what would improve it.

Although a small number of patients recruited into the wider HoldFAST study in two of the locations had been referred to, and had an ongoing involvement with, specialist palliative care services, we were not able to interview any members of those teams. While our research team included clinicians, social scientists and health services researchers, no research team member had a specialist background in palliative care. The clinicians we interviewed were either part of heart failure teams or were experienced in caring for these patients. For that reason, some of the views we encountered might not be representative of those of clinicians more generally. This limitation echoes the perception that qualitative research is not generalisable in the same way as is quantitative research and that findings may not be applicable elsewhere. However, we believe that transparency around our sample, method and results can enable the reader to judge the relevance of our research to their own setting [23].

## Conclusions

Our study highlights the considerable challenges which remain in providing EoL care to heart failure patients. The difficulty in recognising when heart failure patients are approaching the terminal phase of their condition remains a barrier to planning personalised EoL care in advance. There is also a lack of reliable clinical criteria and prognostic models to guide clinicians’ decision-making and inform a transition from active management to palliative care.

While there are increased links between heart failure teams and specialist palliative care services, the HCPs recognised the need for greater sharing of expertise. Specialist heart failure nurses are already co-ordinating services for patients at their end of life and providing generalist palliative care, and would welcome further education and training in this area. Similarly, specialist palliative care services may benefit from the opportunity to learn more about heart failure. Further research should focus on the evaluation of specialist palliative care services for heart failure patients and include patient-centred outcomes.
